# Continuous insulin therapy *versus* multiple insulin injections in the management of type 1 diabetes: a longitutinal study

**DOI:** 10.1016/j.rppede.2015.06.019

**Published:** 2016

**Authors:** Maria Estela Bellini Ribeiro, Raphael Del Roio Liberatore, Rodrigo Custodio, Carlos Eduardo Martinelli

**Affiliations:** Universidade de São Paulo (USP), Ribeirão Preto, SP, Brazil

**Keywords:** Type 1 diabetes mellitus, Insulin, Insulin infusion systems, Adolescents

## Abstract

**Objective:**

To compare multiple doses of insulin and continuous insulin infusion therapy as treatment for type 1 diabetes mellitus.

**Methods:**

40 patients with type 1 diabetes mellitus (21 female) with ages between 10 and 20 years (mean=14.2) and mean duration of diabetes of 7 years used multiple doses of insulin for at least 6 months and after that, continuous insulin infusion therapy for at least 6 months. Each one of the patients has used multiple doses of insulin and continuous insulin infusion therapy. For analysis of HbA1c, mean glycated hemoglobin levels (mHbA1c) were obtained during each treatment period (multiple doses of insulin and continuous insulin infusion therapy period).

**Results:**

Although mHbA1c levels were lower during continuous insulin infusion therapy the difference was not statistically significant. During multiple doses of insulin, 14.2% had mHbA1c values below 7.5% *vs*. 35.71% while on continuous insulin infusion therapy; demonstrating better glycemic control with the use of continuous insulin infusion therapy. During multiple doses of insulin, 15–40 patients have severe hypoglycemic events *versus* 5–40 continuous insulin infusion therapy. No episodes of ketoacidosis events were recorded.

**Conclusions:**

This is the first study with this design comparing multiple doses of insulin and continuous insulin infusion therapy in Brazil showing no significant difference in HbA1c; hypoglycemic events were less frequent during continuous insulin infusion therapy than during multiple doses of insulin and the percentage of patients who achieved a HbA1c less than 7.5% was greater during continuous insulin infusion therapy than multiple doses of insulin therapy.

## Introduction

Diabetes mellitus (DM) is a chronic metabolic syndrome characterized by intense catabolism. Type 1 diabetes mellitus (T1DM) is due to deficient insulin secretion, in most cases after autoimmune destruction of pancreatic beta cells. It is a very frequent chronic disease affecting children,^1^ with incidence increasing all over the world.^2^ In this way, diabetic ketoacidosis (DKA) and hypoglycemia are acute complications of T1DM associated with variety adverse effects and both can have fatal effects if not reversed in time.^3^

The DCCT study (The Diabetes Control and Complications Trial Research Group)^4^ demonstrated that intensive insulin therapy with multiple doses of insulin (MDI) or with a continuous insulin infusion therapy (CIIT) would be the best treatment for T1DM. In spite of the knowledge about both, MDI^5^^–^ 7 and CIIT,^7^ the comparison between these therapeutic schemes, particularly among children and adolescents, is incipient. Several studies have suggested that CIIT may provide better glycemic control,^8^^–^ 11 with lower risk of severe hypoglycemia and a smaller weight gain^8^ compared to the MDI therapy. Among these analyses, few of these studies have been conducted on children and adolescents.^9^^,^ 10

The aim of the present study was to assess, in a comparative manner, the MDI therapy and the use of CIIT regarding metabolic control and the occurrence of acute complications of the disease in a sample of children and adolescents with T1DM followed up at a public hospital in São Paulo state, Brazil.

## Method

This was a longitudinal study based on data obtained retrospectively from the medical records of patients of both sexes aged 5–20 years with a diagnosis of T1DM according to the International Society for Pediatric and Adolescent Diabetes criteria (ISPAD, 1995).

The patients had been using MDI for at least 6 months, and later CIIT, using both brands available in Brazil, also for at least 6 months. The duration of diabetes was required to be more than 2 years.

All patients were trained to count carbohydrates, to modify insulin dosage and to measure capillary glucose levels 7–9 times daily. They all have a telephone number to make contact if they need, and the consultations were made every 3 months by a multi professional team.

The following data were obtained: sex, age, time of MDI use, time of CIIT use, mean glycated hemoglobin levels (mHbA1c), number of severe hypoglycemic events requiring help for recovery, and number of DKA episodes. HbA1c levels were measured by HPLC and it was the same method during all the study.

The research protocol was approved by the Research Ethics Committee with waiver of informed consent.

Data are reported as mean (±SD) and median. The nonparametric Wilcoxon test was used for the paired values of the variables, with the level of significance set at *p*<0.05.

## Results

We analyzed the medical records of 40 patients, 46.4% of them males, who first used MDI and later CIIT during the period from 2011 to 2012. At the time of data collection, patient's age ranged from 10 years and eight months to 20 years and 2 months (mean±standard deviation: 14±2.35 years). Time since the diagnosis of the disease ranged from 2 years and 2 months to 15 years and 3 months (mean 7.0 years).

Time of MDI ranged from 8 months to 14 years and 9 months (mean±standard deviation: 5.1±3.6 months). Time of CIIT use ranged from 6 months to 4 years and 5 months (mean±standard deviation: 1.4±3.6 years) (Table 1).

**Table 1:** Mean glycated hemoglobin (mHbA1c) values (%) for the total sample (T), Mean glycated hemoglobin for patients who were submitted to at least 1 year of each treatment (1y) and 2 years of each treatment (2y).

mHbA1c (n=40)	Mean±SD	Median	*p* -value a
MDI (T)	9.1±2.0	8.6	0.55 b
CIIT (T)	8.9±2.5	8.2	
MDI (1 y)	9.3±2.3	8.6	0.095 c
CIIT (1 y)	8.4±1.9	7.8	
MDI (2 y)	8.4±0.9	8.4	0.67 d
CIIT (2 y)	8.4±2.3	7.7	

a Single-sample nonparametric Wilcoxon test.

b Comparison between MDI and CIIT.

c Comparison between MDI and CIIT (1y).

d Comparison between MDI and CIIT (2y).

SD, standard deviation; MDI, multiple doses of insulin; CIIT, continuous insulin infusion therapy.

The data did not show variation according to sex.

For analysis of HbA1c levels, the results obtained during each treatment period (MDI and CIIT) were retrieved and the mHbA1c value was calculated for each patient and each period. The mean and the median mHbA1c values were then calculated for the sample and for the period of each treatment. Table 2 shows the mHbA1c results obtained in the two periods. The data show that, although mHbA1c levels were lower during the use of CIIT than during the use of MDI, no significant difference was observed.

**Table 2:** Mean glycated hemoglobin (mHbA1c) values (%) for patients with levels of less than 7.5% during only one of the treatments.

Patient	mHbA1c during MDI	mHbA1c during CIIT
1	7.85	7.1
2	7.96	7.33
3	7.9	6.87
4	9.45	7.26
5	8.33	7.26
6	8.4	5.35
7	8.61	7.5
8	9.3	7.05
9	7.1	7.5
10	7.06	7.7
11	7.1	7.8
12	7.1	7.5

mHba1c, mean glycated hemoglobin for each patients; MDI, multiple daily insulin injections; CIIT, continuous insulin infusion therapy.

Analysis of the data for patients who had used at least one year of each treatment revealed that mHbA1c levels were 9.3 (±2.3)% during the MDI period (median: 8.6), and 8.4 (±1.9)% during the CIIT period, with a median of 7.8% (*p*=0.095), showing that there was no significant difference in mHbA1c levels obtained during the two treatments (Table 1).

Analysis of the data for patients who had used at least two years of each treatment revealed that mHbA1c levels were 8.4 (±0.9%) during the MDI period, with a median of 8.4, and 8.4 (±2.3%) with a median of 7.7% during the period of use of CIIT. Again, the Wilcoxon test showed no significant difference between periods (*p*=0.67) (Table 1).

During the use of MDI, 14.2% had mHbA1c values below 7.5%, the value recommended by ISPAD as target to metabolic control, and during the use of CIIT 35.71% had mHbA1c values below 7.5%, demonstrating better glycemic control with the use of an infusion pump for treatment.

Analysis of cases with less than 7.5% of mHbA1c levels in only one of the treatments (MDI or CIIT) revealed that eight of the 12 patients (66.6%) showed reduced mHbA1c levels when they switched to CIIT, while four patients (33.3%) showed lower mHbA1c levels during treatment with MDI (Table 2).

Regarding acute complications, the number of hospitalizations and visits to emergency services due to hypoglycemic and diabetic ketoacidosis events were recorded during the two treatment periods.

During MDI, 15–40 patients have hypoglycemic events needing help from other person. From those 15 patients, 3 had two events, 1 tree events and one, 4 events. No ketoacidosis events were recorded.

During CIIT, 5–40 patients have hypoglycemic events needing help from other person. From those 5, only one event by each one was recorded. Again, no ketoacidosis events were recorded.

Analysis of the mean number of events showed that more complications occurred during MDI treatment than during CIIT (*p*=0.021, single-sample nonparametric Wilcoxon test).

## Discussion

HbA1c level is the most useful measure for the evaluation of metabolic control and the only one demonstrating good correlation with vascular complications.^12^^–^ 14 The process of hemoglobin glycation involves a permanent bond with reducing sugars such as glucose and, because of this irreversible bond, HbA1c values correspond to the hyperglycemia levels of DM patients in a more reliable and true manner than the fasting glycemic levels.

The DCCT study demonstrated that, when HbA1c exceeds 7.5%, the risk of complications increases significantly.^12^^–^ 14 The ISPAD^15^ recommends a value of HbA1c of less that 7.5%.

The present study showed that 14.2% of the patients had lower than 7.5% mHbA1c during the use of MDI, while 35.71% showed an mHbA1c value lower than 7.5% with the use of CIIT, demonstrating better glycemic control with the use of infusion pump therapy. The same conclusion was observed when individual analysis cases of patients with mHbA1c lower than 7.6% in at least one of the treatments (Table 2).

Comparison of the periods of use of MDI and CIIT revealed a reduction of both the mean (9.1–8.9) and the median (8.6–8.2) values of HbA1c levels; although these values no significant difference was observed. Several studies, especially those conducted on young individuals, have demonstrated that CIIT and MDI induce similar results of glycemic control.^16^^–^ 19 In a recent meta-analysis, Yeh et al.^16^ analyzed 33 randomized and controlled studies comparing the two therapies in children and adults with T1DM and concluded that most studies showed similar effects on the control of glycemia in children, with a favorable effect of the use of CIIT on the reduction of HbA1c levels in adults. However, other studies have shown that the use of CIIT promotes a reduction of HbA1c levels both in children^20^^–^ 24 and in adults.^21^^,^ 22 ^,^ 24

In view that the time of use of one of the therapies might influence the evaluation of HbA1c levels, the same analysis of mean and median HbA1c values were performed in patients with at least 1 year of each treatment. Another analysis was performed in patients with at least 2 years of each treatment (Table 1). However, no significant difference was detected favoring one of the treatments.

Regarding adverse effects, a systematic review by Pickup et al.^25^ showed that the frequency of severe hypoglycemia episodes was reduced 4.2 times with the use of CIIT, compared to MDI, although other studies did not detect a significant difference in the number of adverse events between the two treatments.^20^^–^ 22 ^,^ 26 In the present patient series, there was a reduction of the frequency of severe hyperglycemic episodes. Similarly to our data, a previous Brazilian study has also showed reduction of severe hypoglycemia episodes. In spite of fewer subjects and shorter period of observation, this analysis showed a little improvement of metabolic control; furthermore, the authors have not compared MDI and CITT.^27^ A recent paper showed in 345 patients a 0.6% reduction of HbA1c levels from injections to insulin pump therapy. The authors also showed a reduction of severe hypoglycemic events and DKA episodes.^28^

In the design of the present study, the fact that each patient acted as his own control eliminated inter individual differences (such as eating habits, patterns of physical activities, motivation and attitude toward the disease, among others) that might interfere with the analysis since two samples, even when matched, may show differences. However, a limitation of the study is the fact that it was not controlled, with a retrospective and with small sample. Other important issue is the period of observation; probably longer periods of time could have shown a significant difference in the metabolic control. Despite these limitations, to the best of our knowledge, this is the first study with this design comparing the use of two forms of basal-bolus therapy for the metabolic control of diabetes and of the occurrence of acute complications of the disease, in Brazil.

In conclusion, intensive insulin therapy (MDI or CIIT) represents the best form of treatment in order to obtain adequate metabolic control for T1DM patients. Analysis of several studies showed that there is no consensus about the choice between MDI and CIIT for the treatment of T1DM, regarding glycemic control and the rates of adverse events. The present study is the first in Brazil to compare the two forms of therapy with a design of patient as self-control, showing improvement in metabolic control with the use of CIIT, with a reduced occurrence of acute complications of diabetes in this sample.
