# Supplementary material for: Continuous insulin therapy versus multiple insulin injections in the management of type 1 diabetes: a longitutinal study
Source: Rev Paul Pediatr. 2016 Jan-Mar;34(1):86–90. doi: 10.1016/j.rppede.2015.06.019 (PMC4795726; doi:10.1016/j.rppede.2015.06.019)
Supplement: Translation

# Translation: Insulinoterapia contínua versus múltiplas injeções de insulina no tratamento da diabetes tipo 1: um estudo longitudinal

**Authors:** Maria Estela Bellini Ribeiro, Raphael Del Roio Liberatore, Rodrigo Custodio, Carlos Eduardo Martinelli

**Affiliations:** Universidade de São Paulo (USP), Ribeirão Preto, SP, Brasil

**Keywords:** Diabetes melito tipo 1, Insulina, Sistemas de infusão de insulina, Adolescentes

## Abstract

**Objetivo:**

Comparar terapia com múltiplas doses de insulina e o sistema de infusão continua de insulina no tratamento da diabetes melito tipo 1.

**Métodos:**

40 pacientes com diabetes melito tipo 1 (21 mulheres) com idades entre 10 e 20 anos (média=14,2) e duração média do diabetes de sete anos utilizaram múltiplas doses de insulina durante pelo menos seis meses e, depois disso, sistema de infusão continua de insulina por pelo menos seis meses. Todos os pacientes usaram múltiplas doses de insulina e sistema de infusão continua de insulina. Para a análise de HbA1c, níveis médios de hemoglobina glicada (mHbA1c) foram obtidos em cada período de tratamento (múltiplas doses de insulina e sistema de infusão continua de insulina).

**Resultados:**

Embora os níveis de mHbA1c tenham sido menores com o uso de sistema de infusão continua de insulina a diferença não foi estatisticamente significante. Durante o uso de múltiplas doses de insulina, 14,2% tiveram valores de mHbA1c <7,5% vs. 35,71% quando usando sistema de infusão continua de insulina; demonstrando melhor controle glicêmico com o uso de sistema de infusão continua de insulina. Durante o uso de múltiplas doses de insulina, 15-40 pacientes tiveram eventos hipoglicêmicos graves contra 5–40 com sistema de infusão continua de insulina. Não foram registrados episódios de cetoacidose.

**Conclusões:**

Esse é o primeiro estudo cujo desenho comparou o uso de múltiplas doses de insulina e sistema de infusão continua de insulina no Brasil, não demonstrando nenhuma diferença significativa nos níveis de HbA1c. Eventos hipoglicêmicos foram menos frequentes com o uso de sistema de infusão continua de insulina do que com múltiplas doses de insulina e a porcentagem de pacientes que obteve um HbA1c <7,5% foi maior com sistema de infusão continua de insulina do que com múltiplas doses de insulina.

## Introdução

Diabetes melito (DM) é uma síndrome metabólica crônica caracterizada por intenso catabolismo. Diabetes melito tipo 1 (DM1) ocorre devido à secreção deficiente de insulina, na maioria dos casos após a destruição autoimune das células beta pancreáticas. É uma doença crônica muito frequente que afeta crianças,^1^ com uma incidência crescente em todo o mundo.^2^ Dessa forma, a cetoacidose diabética (CAD) e a hipoglicemia são complicações agudas da DM1 associadas com vários efeitos adversos, e ambas podem ter efeitos fatais se não forem revertidas a tempo.^3^

O estudo DCCT (The Diabetes Control and Complications Trial Research Group)^4^ demonstrou que a terapia insulínica intensiva com múltiplas doses de insulina (MDI) ou o sistema de infusão contínua (SIC) de insulina seria o melhor tratamento para a DM1. Apesar do conhecimento sobre ambos, MDI^5^^–^ 7 e SIC,^7^ a comparação entre esses esquemas terapêuticos, especialmente entre as crianças e adolescentes, é incipiente. Vários estudos têm sugerido que o SIC pode proporcionar um melhor controle glicêmico,^8^^–^ 11 com menor risco de hipoglicemia grave, e menor ganho de peso em comparação com a terapia com MDI. Entre essas análises, poucos desses trabalhos foram realizados em crianças e adolescentes.^9^^,^ 10

O objetivo do presente estudo foi avaliar, de forma comparativa, a terapia com MDI e o uso do SIC sobre o controle metabólico e a ocorrência de complicações agudas da doença em uma amostra de crianças e adolescentes com DM1, acompanhados em um hospital público no estado de São Paulo, Brasil.

## Método

Este foi um estudo longitudinal baseado em dados obtidos retrospectivamente dos prontuários de pacientes de ambos os sexos com idade entre 5 e 20 anos com diagnóstico de DM1, de acordo com os critérios da International Society for Pediatric and Adolescent Diabetes (ISPAD, 1995).

Os pacientes usaram MDI por pelo menos 6 meses, e depois o SIC, usando ambas as marcas disponíveis no Brasil, também por pelo menos 6 meses. A duração da diabetes deveria ser maior do que dois anos.

Todos os pacientes foram treinados para contar carboidratos, modificar a dose de insulina e medir os níveis de glicose capilar de 7 a 9 vezes ao dia. Todos eles tinham um número de telefone para fazer contato em caso de necessidade, e as consultas eram feitas a cada 3 meses por uma equipe multiprofissional.

Os seguintes dados foram obtidos: sexo, idade, tempo de uso da terapia com MDI, tempo de uso do SIC, níveis médios de hemoglobina glicada (mHbA1c), número de eventos hipoglicêmicos graves necessitando de ajuda para a recuperação, e número de episódios de CAD. Os níveis de HbA1c foram medidos por cromatografia líquida de alta eficiência (CLAE) e o mesmo método foi adotado durante todo o estudo.

O protocolo de pesquisa foi aprovado pelo Comitê de Ética em Pesquisa com dispensa do consentimento informado.

Os dados são apresentados como média (±DP) e mediana. O teste não paramétrico de Wilcoxon foi utilizado para os valores das variáveis pareadas, com nível de significância de *p*<0,05.

## Resultados

Foram analisados os prontuários de 40 pacientes, 46,4% deles do sexo masculino, que utilizaram primeiro a terapia com MDI e depois o SIC durante o período de 2011 a 2012. No momento da coleta de dados, a idade dos pacientes variou de 10 anos e oito meses a 20 anos e 2 meses (média±desvio padrão: 14±2,35 anos). O tempo desde o diagnóstico da doença variou de 2 anos e 2 meses a 15 anos e 3 meses (média de 7,0 anos).

O tempo de uso da terapia com MDI variou de 8 meses a 14 anos e 9 meses (média±desvio padrão: 5,1±3,6 meses). O tempo de uso do SIC variou de 6 meses a 4 anos e 5 meses (média±desvio padrão: 1,4±3,6 anos) (tabela 1).

**Tabela 1:** Valores médios de hemoglobina glicada (mHbA1c) (%) para a amostra total (T), hemoglobina glicada média para os pacientes que foram submetidos a pelo menos 1 ano de cada tratamento (1a) e 2 anos de cada tratamento (2a)

mHbA1c (n=40)	Média±DP	Mediana	*p* ‐valor a
MDI (T)	9,1±2.0	8,6	0,55 b
SIC (T)	8,9±2.5	8,2	
MDI (1 a)	9,3±2.3	8,6	0,095 c
SIC (1 a)	8,4±1.9	7,8	
MDI (2 a)	8,4±0.9	8,4	0,67 d
SIC (2 a)	8,4±2.3	7,7	

a Teste não paramétrico de Wilcoxon – amostra única.

b Comparação entre MDI e SIC.

c Comparação entre MDI e SIC (1a).

d Comparação entre MDI e SIC (2a).

DP, desvio padrão; MDI, múltiplas doses de insulina; SIC, sistema de infusão contínua de insulina.

Os dados não apresentaram variação de acordo com o sexo.

Para a análise de níveis de HbA1c, os resultados obtidos durante cada período de tratamento (MDI e SIC) foram recuperados e o valor da mHbA1c foi calculado para cada paciente e para cada período. A média e a mediana da mHbA1c foram então calculadas para a amostra e para o período de cada tratamento. A tabela 2 mostra os resultados obtidos da mHbA1c nos dois períodos. Os dados mostram que, embora os níveis de mHbA1c tenham sido menores durante a utilização do SIC do que durante a utilização do MDI, não foi observada diferença significativa.

**Tabela 2:** Valores médios de hemoglobina glicada (mHbA1c) (%) para pacientes com níveis inferiores a 7,5% durante apenas um dos tratamentos

Paciente	mHbA1c durante MDI	mHbA1c durante SIC
1	7,85	7,1
2	7,96	7,33
3	7,9	6,87
4	9,45	7,26
5	8,33	7,26
6	8,4	5,35
7	8,61	7,5
8	9,3	7,05
9	7,1	7,5
10	7,06	7,7
11	7,1	7,8
12	7,1	7,5

mHba1c, hemoglobina glicada média para cada paciente; MDI, múltiplas injeções diárias de insulina; SIC, sistema de infusão contínua de insulina.

A análise dos dados para os pacientes que tinham utilizado cada tratamento por pelo menos um ano revelou que os níveis de mHbA1c foram de 9,3 (±2,3)% durante o período com MDI (mediana: 8,6), e 8,4 (±1,9)% durante o período com SIC, com uma mediana de 7,8% (*p*=0,095), indicando que não houve nenhuma diferença significativa nos níveis de mHbA1c obtidos durante os dois tratamentos (tabela 1).

A análise dos dados para os pacientes que tinham utilizado cada tratamento por pelo menos dois anos revelou que os níveis de mHbA1c foram de 8,4 (±0,9%) durante o período de MDI, com mediana de 8,4, e 8,4 (±2,3%), e mediana de 7,7%, durante o período de utilização do SIC. Mais uma vez, o teste de Wilcoxon não mostrou nenhuma diferença significativa entre os períodos (*p*=0,67) (tabela 1).

Durante a utilização de MDI, 14,2% dos pacientes apresentaram valores de mHbA1c abaixo de 7,5%, valor recomendado pelo ISPAD como a meta para o controle metabólico, e durante a utilização do SIC, 35,71% apresentaram valores de mHbA1c abaixo de 7,5%, o que demonstra um melhor controle glicêmico com o uso de uma bomba de infusão para o tratamento.

A análise dos casos com menos de 7,5% nos níveis de mHbA1c em apenas um dos tratamentos (MDI ou SIC) revelou que oito dos 12 pacientes (66,6%) apresentaram níveis de mHbA1c reduzidos quando mudaram para o SIC, enquanto quatro pacientes (33,3%) apresentaram níveis mais baixos de mHbA1c durante o tratamento com MDI (tabela 2).

Quanto às complicações agudas, foram registrados os números de internações e atendimentos nos serviços de emergência devido a eventos hipoglicêmicos e cetoacidose diabética durante os dois períodos de tratamento.

Durante a terapia com MDI, 15 a 40 pacientes apresentaram eventos hipoglicêmicos com necessidade de ajuda de outra pessoa. Desses 15 pacientes, três tiveram dois eventos, 1 três eventos e outro, 4 eventos. Não foram registrados eventos de cetoacidose.

Durante o uso do SIC, 5 a 40 pacientes apresentaram eventos hipoglicêmicos com necessidade de ajuda de outra pessoa. Desses cinco, apenas um evento foi registrado para cada um. Mais uma vez, não foram registrados eventos de cetoacidose.

A análise do número médio de eventos mostrou que mais complicações ocorreram durante o tratamento com MDI do que com o SIC (*p*=0,021, teste de Wilcoxon não paramétrico de amostra única).

## Discussão

O nível de HbA1c é a medida mais útil para a avaliação do controle metabólico e o único que demonstra boa correlação com complicações vasculares.^12^^–^ 14 O processo de glicação da hemoglobina envolve uma ligação permanente com açúcares redutores como a glicose e, devido a essa ligação irreversível, os valores de HbA1c correspondem aos níveis de hiperglicemia de pacientes com DM de uma maneira mais confiável e verdadeira do que os níveis glicêmicos de jejum.

O estudo DCCT demonstrou que, quando a HbA1c excede 7,5%, o risco de complicações aumenta significativamente.^12^^–^ 14 O ISPAD^15^ recomenda um valor de HbA1c menor que 7,5%.

O presente estudo mostrou que 14,2% dos pacientes apresentou um valor de mHbA1c menor do que 7,5% durante a utilização de MDI, enquanto 35,71% mostrou um valor mHbA1c inferior a 7,5% com o uso do SIC, demonstrando um melhor controle glicêmico com o uso de bombas de infusão. A mesma conclusão foi observada em análises individuais de casos de pacientes com mHbA1c inferior a 7,6% em pelo menos um dos tratamentos (tabela 2).

A comparação dos períodos de utilização de MDI e SIC revelou uma redução dos níveis de HbA1c tanto nos valores da média (9,1-8,9) quanto da mediana (8,6-8,2); apesar desses valores, não foi observada uma diferença significativa. Diversos estudos, especialmente aqueles realizados em indivíduos jovens, demonstraram que o SIC e a terapia com MDI induzem resultados semelhantes de controle glicêmico.^16^^–^ 19 Em uma meta-análise recente, Yeh et al.^16^ analisaram 33 estudos randomizados e controlados, comparando as duas terapias em crianças e adultos com DM1, e concluíram que a maioria dos estudos mostrou efeitos semelhantes no controle da glicemia em crianças, com um efeito favorável do uso do SIC sobre a redução dos níveis de HbA1c em adultos. No entanto, outros estudos mostraram que a utilização do SIC promove a redução dos níveis de HbA1c tanto em crianças^20^^–^ 24 quanto em adultos.^21^^,^ 22 ^,^ 24

Tendo em conta que o tempo de utilização de uma das terapias possa influenciar a avaliação dos níveis de HbA1c, a mesma análise dos valores da mediana e da média de HbA1c foi realizada em pacientes com pelo menos 1 ano de cada tratamento. Outra análise foi realizada em pacientes com, pelo menos, dois anos de cada tratamento (tabela 1). No entanto, nenhuma diferença significativa foi detectada favorecendo um dos tratamentos.

Com relação aos efeitos adversos, uma revisão sistemática realizada por Pickup et al.^25^ mostrou que a frequência de episódios de hipoglicemia grave foi reduzida 4,2 vezes com o uso de SIC, em comparação com MDI, embora outros estudos não tenham detectado uma diferença significativa no número de eventos adversos entre os dois tratamentos.^20^^–^ 22 ^,^ 26 Na presente série de pacientes houve uma redução da frequência de episódios hiperglicêmicos graves. De forma similar aos nossos dados, um estudo brasileiro anterior também mostrou redução dos episódios de hipoglicemia grave. Apesar do número menor de indivíduos e período de observação mais curto, essa análise mostrou uma pequena melhora do controle metabólico; além disso, os autores não compararam MDI e SIC.^27^ Um estudo recente mostrou em 345 pacientes uma redução de 0,6% dos níveis de HbA1c que mudaram do tratamento com injeções para a bomba de insulina. Os autores também mostraram uma redução de eventos hipoglicêmicos graves e episódios de CAD.^28^

No desenho do presente estudo, o fato de que cada paciente atuou como seu próprio controle, eliminou as diferenças interindividuais (tais como hábitos alimentares, padrões de atividade física, motivação e atitude em relação à doença, entre outros), que podiam interferir com a análise, já que duas amostras, mesmo quando pareadas, podem apresentar diferenças. No entanto, uma limitação do estudo é o fato de que ele não foi controlado, com uma amostra retrospectiva e pequena. Outra questão importante é o período de observação; provavelmente períodos de tempo mais longos poderiam ter mostrado uma diferença significativa no controle metabólico. Apesar dessas limitações, que seja de nosso conhecimento, este é o primeiro estudo com esse desenho comparando o uso de duas formas de terapia basal-bolus para o controle metabólico da diabetes e a ocorrência de complicações agudas da doença, no Brasil.

Em conclusão, a terapia intensiva de insulina (com MDI ou SIC) representa a melhor forma de tratamento com o objetivo de obter o controle metabólico adequado para pacientes com DM1. A análise de vários estudos mostrou que não há consenso sobre a escolha entre MDI e SIC para o tratamento da DM1, em relação ao controle glicêmico e às taxas de eventos adversos. O presente estudo é o primeiro no Brasil a comparar as duas formas de terapia com um desenho utilizando o próprio paciente como autocontrole, mostrando melhora do controle metabólico com o uso de SIC, com uma menor ocorrência de complicações agudas da diabetes nesta amostra.
